# Prognostic impact of tumor volume in patients with complete resection of thymoma

**DOI:** 10.1111/1759-7714.14353

**Published:** 2022-02-15

**Authors:** Yudai Miyashita, Takashi Kanou, Hiroto Ishida, Eriko Fukui, Naoko Ose, Soichiro Funaki, Masato Minami, Yukihisa Sato, Masahiro Yanagawa, Yasushi Shintani

**Affiliations:** ^1^ Department of General Thoracic Surgery Osaka University Graduate School of Medicine Suita Japan; ^2^ Department of Radiology Osaka University Graduate School of Medicine Suita Japan

**Keywords:** recurrence, thymoma, tumor volume

## Abstract

**Background:**

The impact of tumor volume on prognosis is unclear. We therefore investigated the correlation between tumor volume and prognosis in patients with complete resection of thymoma.

**Methods:**

A total of 177 patients who underwent curative surgical resection for thymoma were retrospectively collected and reviewed. We performed a volumetric analysis of each case using the modified version of “Watchin GGO” and evaluated the relationship between tumor volume and recurrence.

**Results:**

The median tumor size was 5.0 (range 0.5–15) cm, and the median tumor volume was 35.1 (range 0.265–881.0) cm^3^. The Pearson product–moment correlation coefficient was 0.658, suggesting a moderately strong connection between tumor volume and tumor size. To determine the impact of tumor volume on tumor recurrence, receiver operating characteristic curves of the recurrence and tumor volume were calculated. The area under the curve was 0.65 (95% confidence interval [CI]: 0.51–0.80), and the optimal cutoff level of the tumor volume for recurrence was 82.6 cm^3^, with a sensitivity and specificity of 0.64 (11/17) and 0.74 (119/160), respectively. Patients with tumors ≥82.6 cm^3^ had a significantly worse recurrence‐free survival than those with smaller tumors (*p* = 0.0122, hazard ratio: 2.99), with 5‐year recurrence rates of 74.9% (95% CI: 58.6%–86.3%) versus 88.9% (95% CI: 79.0%–94.4%).

**Conclusion:**

The volume of completely resectable thymoma may be a useful prognostic indicator.

## Graphical abstract

We conducted the tumor volume analysis and investigated the correlation between the volume and the prognosis after complete resection for thymoma. Patients with tumors ≥82.6 cm^3^ showed a significantly worse recurrence‐free survival than those with smaller tumors. The volume of thymoma may be a useful prognostic indicator for the recurrence after surgical treatment.

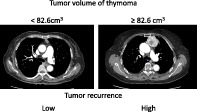

## INTRODUCTION

The Masaoka classification and tumor‐node‐metastasis (TNM) classification from IASLC/ITMIG are widely recognized staging systems for thymic epithelial tumors.^1^, ^2^, ^3^, ^4^ These systems emphasize the presence of invasion into the adjacent organs for tumor staging. While the tumor size has been shown to be a prognostic factor for the recurrence of thymoma in previous reports,^5^, ^6^, ^7^, ^8^, ^9^ the cutoff values have varied. More sensitive and accurate indicators are thus required as novel prognostic factors.

With improvements in image analysis technology, the relationship between tumor volume and prognosis has been reported in the lung cancer research field.^10^ It has previously been considered difficult to precisely measure the volume of a thymoma. Recently, the modified version of “WatchinGGO” (LISIT), a software program for measuring the volume of ground‐glass opacity shadows, has been reported as a promising method for precisely measuring the volume of thymic epithelial tumors.^11^

In the present study, we investigated the relationship between tumor volume and postoperative recurrence of thymoma. To achieve this goal, we used an image analysis software program to measure thymoma volume and analyze its impact on the rate of recurrence after surgical resection.

## METHODS

### Patients

Between 2007 and 2017, 184 consecutive patients with thymoma, including stage IV disease, received surgical treatment in our institution. Seven patients were excluded due to incomplete R2 resection, and 177 patients were ultimately enrolled in this study. The clinicopathological features and outcomes of the treatment were retrospectively investigated. The study protocol was approved by the Ethics Review Board for Clinical Studies of Osaka University (control no. 18297).

The following factors were collected from the medical records of each patient: gender, age, tumor size, presence of pre‐ or postoperative treatment, autoimmune diseases, surgical approach, extent of resection, pathological stage, and date of follow‐up. Tumor staging was performed in accordance with the Masaoka stage and IASLC/ITMIG stage.^2^, ^12^ Based on the WHO classification, the tumors were divided into five subgroups: A, AB, B1, B2, and B3.^13^ Recurrence was diagnosed based on the radiological findings in compliance with the recommendation of the International Thymic Malignancy Interest Group.^14^

### Tumor size and volume

Tumor size was decided based on the maximum diameter described in the radiological report of computed tomography (CT) prior to any treatment. Volumetric analyses of each case were performed using the modified “Watchin GGO” software program as previously reported.^11^ In brief, thin‐section chest CT was performed in all cases, and preoperative CT images were reviewed retrospectively. Volumetric measurements were semi‐automatically obtained using the above software program. The role of Y.M. (the author) in the volumetric measurement was to select the tumor and roughly trace it with a cursor on a single CT slice. We also reported that this software program has accuracy in interobserver agreement and repeatability of measurements.

### Statistical analysis

Relationship through the Pearson product–moment correlation coefficient was used. The recurrence‐free survival (RFS) was determined as the period from the date of surgery to that of the first recurrence after radical complete resection. The Kaplan–Meier method was used to describe the survival curve and survival rate. Statistical differences between survival curves were examined by a log‐rank test. Cox's proportional hazards model was utilized to decide the independent risk factors for the recurrence. The Mann–Whitney U test was used to compare the differences between two independent samples. The predictive validity was compared using the receiver operating characteristic (ROC) curve. The area under the curve (AUC) was measured to investigate the screening power of the tumor size and volume for the prediction of recurrence. The optimal cutoff values were decided by the scores of the sensitivity and specificity at various cutoff scores. Y.M. (the author) performed all statistical analyses with the JMP pro 15 software program (SAS Institute Inc).

## RESULTS

### Patient characteristics

Between 2007 and 2017, 184 patients underwent surgical treatment for thymoma. Of those 184 patients, the 177 who underwent complete surgical resection were enrolled in this study. The follow‐up period ranged from 2 to 134 months, with a median of 61 months. The entire cohort included 68 (38.4%) men and 109 (61.6%) women, with a median age of 56 (range 23–83) years old. The patient distribution of IASLC/ITMIG stage and Masaoka stage are described in Table 1. The number in each WHO classification was distributed as follows: type A,^4^ AB (41), B1 (57), B2 (50), and B3 (25). Induction therapy was performed for 23 patients. Open thoracotomy was performed in 103 patients, and a video‐assisted thoracic surgery (VATS) or robot‐assisted thoracic surgery (RATS) approach was utilized in 74 patients.

**TABLE 1:** Patient characteristics

Age, years	
Median (range)	56 (23–83)
Sex (%)	
Female	109 (61.6)
Male	68 (38.4)
WHO classification (%)	
A	4 (2.3)
AB	41 (23.2)
B1	57 (32.2)
B2	50 (28.2)
B3	25 (14.1)
Masaoka stage (%)	
I	114 (64.4)
II	33 (18.6)
III	15 (8.5)
IV	15 (8.5)
IASLC/ITMIG stage (%)	
I	147 (83.0)
II	3 (1.7)
III	12 (6.8)
IV	15 (8.5)
Tumor size	
Median (range), cm	5.0 (0.5–15.0)
Tumor volume	
Median (range), cm^3^	35.1 (0.3–881.0)
Procedure (%)	
Partial resection	49 (27.7)
Thymectomy	22 (12.4)
Extended thymectomy	106 (59.9)
Surgical approach (%)	
Open	105 (59.3)
VATS/RATS	71 (40.1)
Induction therapy	
Chemotherapy	22
Chemoradiotherapy	1
Adjuvant therapy	
Radiotherapy	1
Myasthenia gravis (%)	
(+)	65 (36.7)
(−)	112 (63.3)
Recurrence (%)	
(+)	17 (9.6)
(−)	160 (90.4)

Abbreviations: IASLC/ITMIG, the International Association for the Study of Lung Cancer and the International Thymic Malignancy Interest Group; RATS, robot‐assisted thoracic surgery; VATS, video‐assisted thoracic surgery; WHO, World Health Organization.

### Relationship between tumor size and tumor volume

**FIGURE 1:**
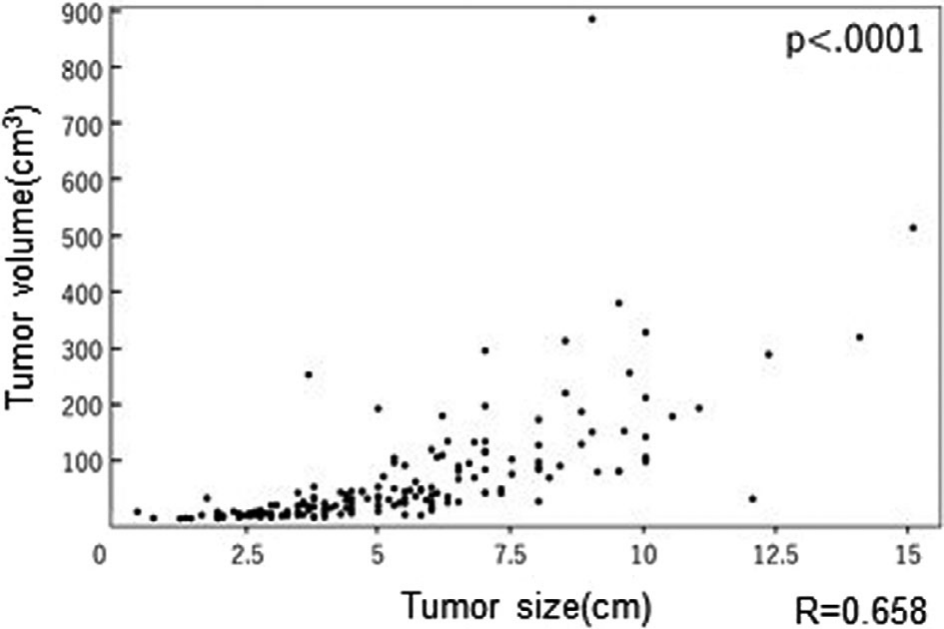
The relationship between tumor size and volume

The relationship between tumor size and volume is shown in Figure 1. The median tumor size was 5.0 (range 0.5–15) cm, and the median tumor volume was 35.1 (range 0.265–881.0) cm^3^. The Pearson product–moment correlation coefficient was 0.658, suggesting a moderately strong connection between the tumor volume and size (*p* < 0.0001).

### Patient distribution in accordance with tumor volume

**FIGURE 2:**
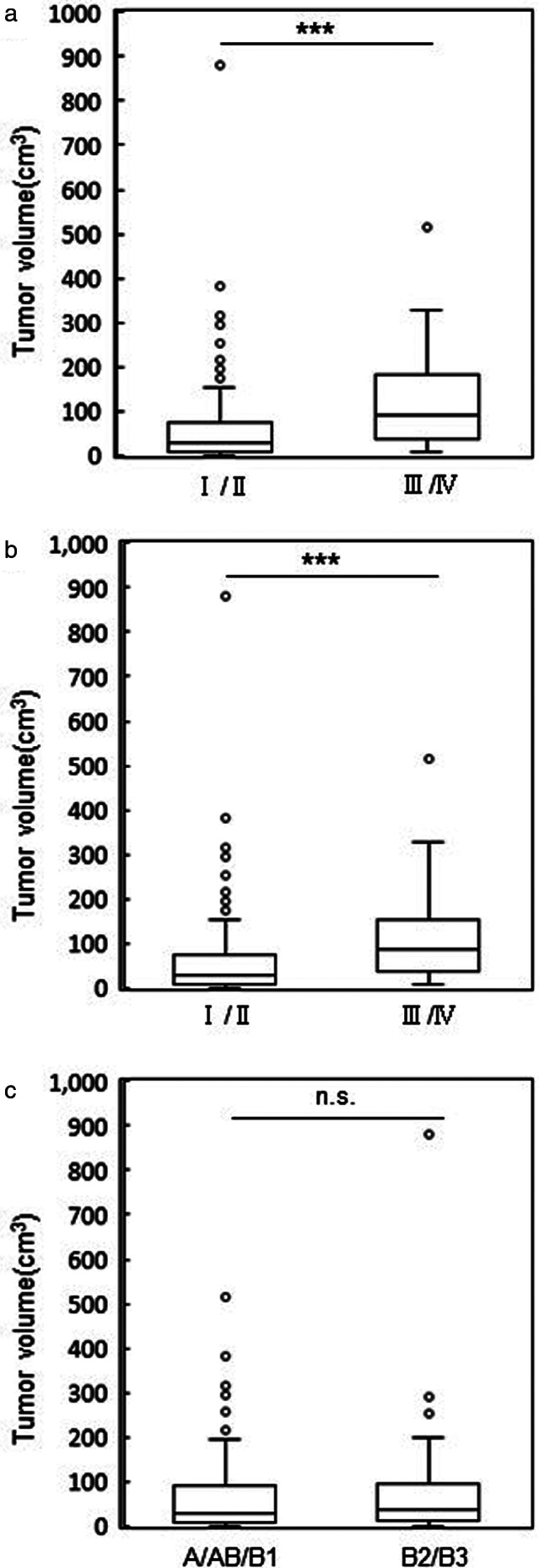
(a) The comparison of tumor volume between the IASLC/ITMIG stage. (b) Comparison of tumor volume between the Masaoka stage. (c) Comparison of tumor volume between the WHO histological classification. ***p < 0.001

The patient distribution according to the tumor volume divided by the IASLC/ITMIG stage, Masaoka stage, and WHO classification are shown in Figure 2. The tumor volume of IASLC/ITMIG stage I and II lesions (range 0.3–881.0 cm^3^, median 29.4 cm^3^) was significantly lower than that in stage III and IV lesions (range 9.3–512.7 cm^3^, median 92.4 cm^3^) (*p* < 0.0001). The tumor volume of Masaoka stage I and II lesions (range 0.3–881.0 cm^3^, median 29.4 cm^3^) was also significantly lower than that of stage III and IV lesions (range 7.1–512.7 cm^3^, median 83.5 cm^3^) (*p* = 0.0003). In contrast, no significant difference in the tumor volume was observed according to the WHO classification of type A/AB/B1 (range 0.3–512.8 cm^3^, median 29.6 cm^3^) and B2/B3 (range 1.1–881.0 cm^3^, median 36.1 cm^3^) (*p* = 0.4499).

### RFS outcomes

**FIGURE 3:**
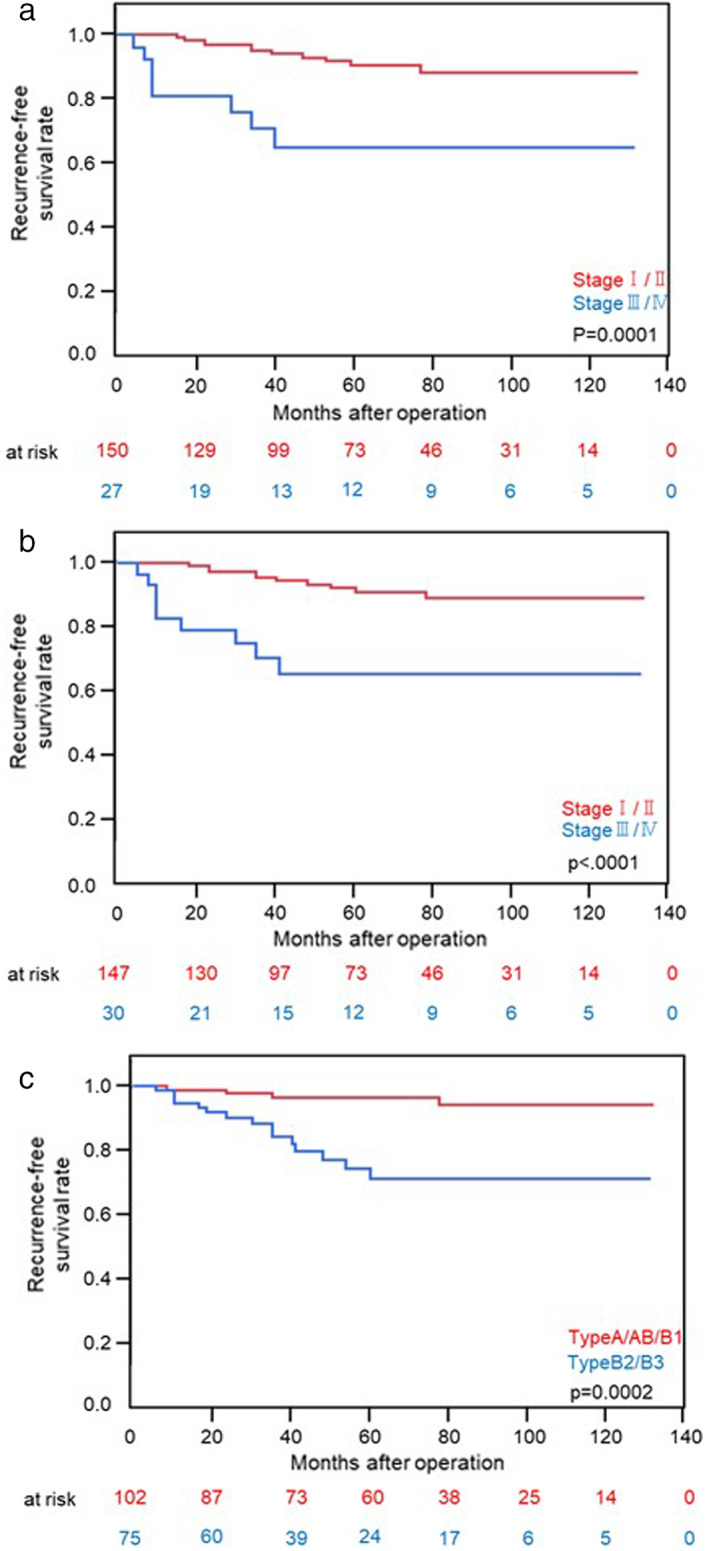
(a) Recurrence‐free survival after complete surgical resection according to the IASLC/ITMIG stage. (b) Recurrence‐free survival after complete surgical resection according to the Masaoka stage. (c) Recurrence‐free survival after complete surgical resection according to the WHO histological classification

Tumor recurrence was confirmed in 17 patients. The 5‐year RFS rate was 85.0% in our entire cohort. The RFS rates according to the IASLC/ITMIG stage, Masaoka stage, and histology based on the WHO classification are shown in Figure 3. These clinicopathological classifications were shown to predict recurrence after radical tumor resection.

### Relationship between the tumor volume and recurrence

**FIGURE 4:**
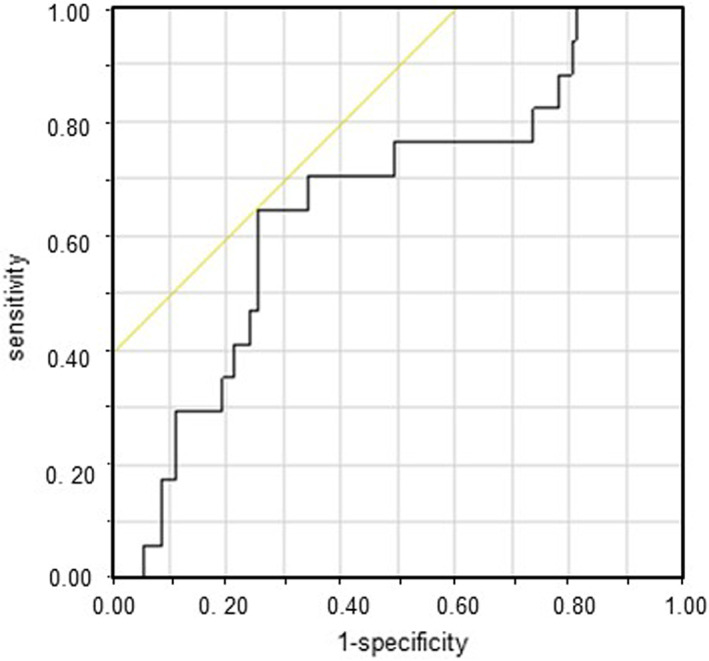
The area under the receiver operating characteristic curve for recurrence determined using tumor volume

To determine the impact of the tumor volume on recurrence, the ROC curve of the recurrence and tumor volume was determined (Figure 4). The AUC was 0.65 (95% confidence interval [CI]: 0.51–0.80), and the optimal cutoff value of the tumor volume for recurrence was 82.6 cm^3^, with a sensitivity and specificity of 0.64 (11/17) and 0.74 (119/160), respectively.

### Predictors of recurrence

**FIGURE 5:**
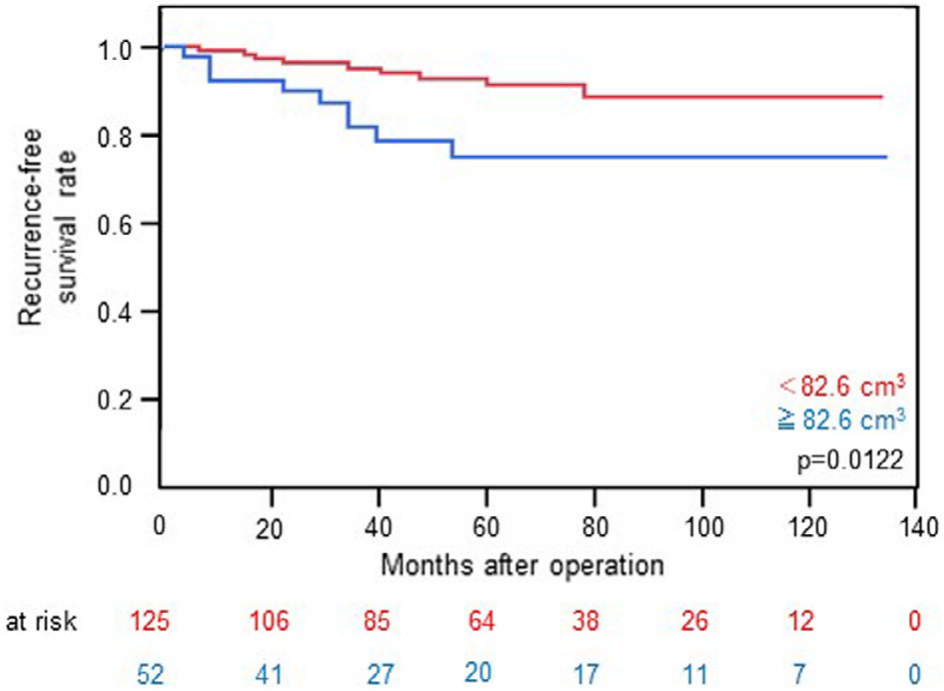
Recurrence‐free survival after complete surgical resection according to tumor volume

A univariate analysis for predictors of the RFS was performed to investigate the relationship between the prognosis and clinical factors, including tumor volume (Table 2). The RFS curve after complete resection in relation to the tumor volume is shown in Figure 5 (*p* = 0.0122). Patients with tumors ≥82.6 cm^3^ had a significantly worse recurrence‐free survival than those with smaller tumors (*p* = 0.0122, hazard ratio: 2.99), with 5‐year recurrence rates of 74.9% (95% CI: 58.6%–86.3%) versus 88.9% (95% CI: 79.0%–94.4%). Furthermore, multivariate analyses for the RFS indicated that a larger tumor volume and higher pathological stage were independent prognostic factors for tumor recurrence (Table 2). A survival analysis revealed that the tumor volume was significantly related to the prognosis after surgical treatment for thymoma.

**TABLE 2:** Results of univariate and multivariate analysis

Variables	Univariate			Multivariate		
	HR	95% CI	*p*‐value	HR	95% CI	*p*‐value
Thoracotomy	2.34	0.788–8.59	0.369			
Volume (≥82.6 cm^3^)	5.32	1.85–15.2	0.0122	2.81	1.07–7.38	0.0359
Size (≥5.0 cm)	2.51	0.960–7.77	0.0664			
Masaoka stage (III/IV)	10	3.41–29.2	<0.0001	2.04	0.728–5.71	0.174
WHO classification (B2, B3)	7.57	2.09–27.4	0.0002	5.24	1.56–17.5	0.0072

Abbreviations: CI, confidence interval.; HR, hazard ratio; WHO, World Health Organization.

## DISCUSSION

In this study, our hypothesis was that there was a correlation between thymoma volume and recurrence rate in surgically‐treated cases. Our findings in the patients registered at our hospital revealed that the incidence of recurrence was significantly higher in patients with thymoma volumes of ≥82.6 cm^3^ than in those with smaller lesions. We also showed that tumor volume was an independent prognostic factor for tumor recurrence among several clinicopathological factors. In the multivariate analysis for the RFS, the Masaoka stage was not an independent prognostic factor. The Masaoka stage was thought to be an independent factor, but in our study, its correlation with the tumor volume was so strong that it seems to have lost its relevance as an independent factor.

Measurement of thymoma diameter can be easily performed in clinical practice using preoperative imaging modalities, such as CT and MRI. Although thymoma size is not considered in the T factor, several studies have been published in recent years that have examined thymoma size and its association with prognosis. Fukui et al.^15^ reported the prognostic importance of tumor size in completely resected cases based on their institutional cohort. They found that a tumor size of ≥4.0 cm was an independent prognostic factor for a worse RFS. Previously from our institution, Okumura et al. found that the incidence of recurrence was significantly higher in patients with thymomas greater than 5.0 cm, and the incidence of tumor death was higher in patients with thymomas greater than 8.0 cm, according to findings obtained from cases registered in the JART database.^9^ They also showed that tumor size was an independent prognostic factor, as it was associated with known prognostic factors, such as WHO histology, pathological stage, and completeness of resection. However, a tumor size of ≥5.0 cm, as previously reported by Okumura et al., did not show a significant impact on the rate of tumor recurrence in our institution.

Previous reports mainly focused on the two‐dimensional maximum diameter of thymoma. Since thymoma does not have a constant shape based on its occupation site, precisely evaluating the maximum tumor diameter seems challenging.^16^ It was previously thought that the tumor volume could be used to evaluate the tumor size more accurately than the two‐dimensional maximum diameter.^10^ Tian et al. found that tumor maximum area may determine the survival outcomes of patients with thymic epithelial tumors.^17^ Using our novel volumetric measurements, the three‐dimensional tumor volume can be measured semi‐automatically by tracing a line around the outer edge of the tumor. However, our search revealed no reports that investigated the relationship between thymoma volume and prognosis.

Tumor size is considered a crucial factor in determining the treatment strategy. VATS and RATS for thymic tumors have become more common and are less invasive than a median sternotomy approach.^18^ Nevertheless, controversy persists regarding the indications for minimally invasive approaches. Our previous study showed that a tumor diameter exceeding 5 cm was a risk factor for recurrence after VATS resection for thymoma.^19^ The present findings suggest that tumor volume may be more useful than tumor size for selecting the surgical approach, although this is a subject for further investigation in the future.

Several limitations associated with the present study warrant mention. First, this study had a single‐institution setting and retrospective design, and the sample size was small. For validation, a different patient group collected separately and prospectively will be needed to verify our conclusion. Second, this analysis was based on our custom‐developed software program that enabled us to quantify the tumor volume semi‐automatically. As the calculation was not fully automatic, minor discrepancies among the observers remains of some concern. A further study will be required to strengthen the accuracy of the measurement.

Despite these limitations, our findings nevertheless support the promise of the tumor volume measurement as a method for predicting the prognosis.

In conclusion, we detected a correlation between thymoma volume and recurrence rate in surgically‐treated cases of thymoma. This finding may contribute to determining which surgical procedure to perform in patients with thymoma.

## CONFLICT OF INTEREST

The authors declare no conflicts of interest.
